# Fruit and Vegetable Intake of Females Before, During, and After Introduction of 3 Bundled Food System Interventions in Urban Vietnam and Nigeria

**DOI:** 10.1016/j.cdnut.2023.102050

**Published:** 2023-12-07

**Authors:** Giulia Pastori, Inge D Brouwer, Meike Siemonsma, Hans Verhoef, Le Thi Huong, Thi Thanh Le Xuan, Truong Tuyet Mai, Folake O Samuel, Oluyemisi F Shittu, Toluwalope E Eyinla, Brice Even, Ricardo Hernandez, Mark Lundy, Alan de Brauw, Sigrid Wertheim-Heck, Kate Ambler, Gennifer Meldrum, Amanda De Filippo, Elise F Talsma

**Affiliations:** 1 Department of Global Nutrition, Division of Human Nutrition and Health, Wageningen University and Research, the Netherlands; 2 International Food Policy Research Institute, United States; 3 Institute for Preventive Medicine and Public Health, Hanoi Medical University, Vietnam; 4 National Institute of Nutrition, Vietnam; 5 Department of Human Nutrition and Dietetics, University of Ibadan, Nigeria; 6 The Alliance of Bioversity International and the International Center for Tropical Agriculture, Vietnam and Colombia

**Keywords:** food system, fruit and vegetable intake, diet quality, low- and middle-income countries, innovations, monitoring, acceptability, accessibility, affordability, females

## Abstract

**Background:**

Low fruit and vegetable (FV) intake in low- and middle-income countries, which is associated with noncommunicable diseases and micronutrient deficiencies, requires food system interventions addressing FV accessibility, affordability, and acceptability. Periodic FV intake monitoring during interventions informs progress toward achieving increased intakes and contributes to understanding the effectiveness of these interventions.

**Objectives:**

This study evaluates the trend in FV intake before, during, and after implementation of a set of nutrition-sensitive food system interventions addressing accessibility, affordability, and acceptability to increase FV consumption over a 1-y period in Vietnamese and Nigerian low-income urban and periurban females.

**Methods:**

We used the Diet Quality Questionnaire to assess FV food group consumption among 600 Vietnamese (Hanoi) and 610 Nigerian (Ibadan) females before, during, and after the interventions (Vietnam: July 2020–September 2021; Nigeria: November 2020–December 2021). A FV score was compared between exposure groups with (mixed) count modeling. The trend in consumption of individual FV groups was analyzed with mixed logistic regression.

**Results:**

The FV score was stable over time, and a small increase was observed after the intervention period especially in Nigeria and in urban Vietnam. A decrease in the total score was observed in periurban Vietnam. Fluctuations were detected in the probability of consumption of individual FV groups over time especially within the fruit groups, probably due to seasonal availability. The degree of exposure could not explain differences in FV intake.

**Conclusions:**

We found a marginal increase in the proportion of females consuming FV during the interventions in both countries. The FV score appeared to be a simple, quick, and easy-to-use indicator for monitoring diversity, variety, and consumption.

## Introduction

The intake of fruit and vegetable (FV) is particularly low in low- and middle-income countries where over 80% of the population [1,2] fail to meet the daily intake requirement of 400 g as recommended by the WHO [3]. FV play an important role in preventing micronutrient deficiencies and diet-related noncommunicable diseases [3]. The health benefits are attributed to their high content of essential minerals, vitamins, phytochemicals, and dietary fiber. These nutrients are often deficient in many diets across the globe [4,5]. Low intake of FV is recognized as a risk factor for the global burden of disease and is associated with a risk of cancer, stroke, cardiovascular disease, and all-cause mortality [6]. Improving FV intake is a key strategy for increasing diet quality.

Dietary intakes and choices for FV are driven by complex combinations and interactions of psychosocial, socioeconomic, and environmental factors related to food system activities [[7], [8], [9], [10]]. Accordingly, there is evidence that interventions focusing on the food environment, behavior change communication, subsidies, and taxes are effective strategies to promote FV consumption [11]. Therefore, a food system approach is needed to make healthy foods, such as FV, accessible, affordable, and acceptable to people with the ultimate goal to improve the quality of their diets [12].

With this rationale, the “Fruit and Vegetable Intake in Vietnam and Nigeria” (FVN) project was implemented in the context of urban and periurban Vietnam and Nigeria to increase FV intake among low-income urbanites through a bundle of 3 food system interventions addressing accessibility, affordability, and acceptability of FV. These interventions included diverse retail-level innovations designed and implemented by small-scale FV (in)formal vendors, a client-specific coupon system, and promotional campaigns about the importance of eating FV daily. The interventions were implemented over a 12-mo period in 2020/2021 in purposely selected low-income urban and periurban areas in Hanoi and Ibadan, where FV consumption is low [13,14] and unprivileged females are at higher risk low-quality diets [15]. (In)formal open-air FV vendors were targeted by the interventions because low-income urbanites mainly depend on these more traditional vending structures (B. Even, S. Crawford, O. F. Shittu, M. Lundy, S. Wertheim-Heck, F. O. Samuel, et al., unpublished results, 2023). In urban Hanoi, these structures contribute to 70% of the food intake among low-income populations [16], and across sub-Saharan Africa, traditional markets and informal traders remain the main source of fresh foods for low- to middle-income urbanites [17,18].

Periodic monitoring of FV intake throughout the period of intervention [19] could provide information on progress in achieving increased intakes and contribute to the final evaluation of the interventions to assess whether the planned objectives are being met and contribute to the limited knowledge and understanding of the effectiveness of nutrition interventions [11,20]. The repetitive nature of the data needed in such periodic monitoring asks for a simple, intuitive, replicable, and noninvasive tool and indicator. Thus, the FV score, previously validated by the authors in the FVN project using the Diet Quality Questionnaire (DQQ) [21], captures well the total FV intake and variety among FV food groups [22], and it is a promising tool to provide a preliminary evaluation of the effect of the interventions and comparison across countries.

This study aimed to assess the changes of FV intake and of single FV food groups consumed over the period of the interventions using the FV score derived from the DQQ. It also evaluated the association between FV food group consumption and degree of exposure to the interventions in urban Vietnamese and Nigerian females targeted by the FVN project.

## Methods

### Study population

The participants were females aged 18 to 49 y from low-income households living in Hanoi, Vietnam and Ibadan, Nigeria. Pregnant and lactating females were excluded from the study. In both cities, one urban and one periurban area were selected for the high prevalence of low-income households: Đống Đa and Hà Đông in Hanoi, and Abàeja and Bagadajé in Ibadan. Participants were selected from the lists of households residing in the selected areas that included at least one female aged 18 to 49 y, provided by community health workers in Vietnam and the local project team in Nigeria. Part of the respondents were recruited in 2019, at the beginning of the FVN project, and part in 2020. The reason for the 2 different rounds of recruitment was the large dropout of females after a break of the project imposed by the COVID-19 pandemic. When it became possible to start with the implementation of the interventions, new respondents were selected to replace the dropout using the same selection method. Data analysis for this study comprised only those that stayed in the study and the replacements of the dropouts, excluding those lost to follow-up during the period of the interventions.

### FVN project

The 3 FVN interventions initially aimed at improving *1*) the accessibility by enlarging the diversification of the FV assortment of FV vendors, *2*) the affordability by means of a client-specific coupon system, and *3*) the acceptability through a promotional campaign about the importance of eating FV daily. All country-specific interventions were then further developed based on data on the dietary intake [23] and knowledge, attitude, and practices around FV consumption [24,25] of the study population, barrier analysis [26], product seasonality [27,28], and market assessment [29,30] of the studied areas (Supplemental Tables 1 and 2). Although the first intervention (*1*) was initially envisaged to focus solely on accessibility, in the end, it focused on affordability and acceptability, as a result of the participatory cocreation method employed (B. Even, S. Crawford, O. F. Shittu, M. Lundy, S. Wertheim-Heck, F. O. Samuel, et al, unpublished results, 2023). Different innovations were implemented, such as improved point of sales and product display (Nigeria), improved marketing (Vietnam and Nigeria), delivery of nutritional information to consumers (Vietnam and Nigeria), improved food safety and customer service practices (Nigeria), and set-up of a loyalty card system (Vietnam). This intervention was implemented for 8 mo in both countries.

The second intervention (*2*) consisted of the distribution of coupons of 2 different monetary values (Vietnam: 30,000/60,000 Vietnamese dong; Nigeria: 400/800 Nigerian naira) to purchase a selection of fruit items (8 in Vietnam and 9 in Nigeria) from selected FV vendors. In Vietnam, coupons were delivered to randomly selected sample households on a biweekly basis, first by a delivery service and then by community health workers 2 mo after the project began. In Nigeria, sets of coupons were delivered to randomly selected sample households by project staff on a weekly basis. In both countries, coupons expired 2 wk after they were received by households and could be redeemed at the retail outlets of participating vendors. The coupon intervention lasted 5 mo in both countries.

The third intervention (*3*) involved a series of neighborhood-specific campaigns to promote the importance of adequate daily FV consumption, which were developed and reviewed through a series of 4 cocreation workshops engaging low-income residents from the study areas. In Vietnam, communication materials (pamphlets, posters) focused messaging around the health benefits of FV, variety, seasonality, WHO recommended intake of 400 g/d, food safety, and home production, and they were disseminated by local health centers through social media platforms, market events, training courses, and loudspeaker announcements (Hà Đông only). In Nigeria, messaging in the communication materials (pamphlets, posters, branded merchandize, jingles, dramas, and expert talks) highlighted disease prevention, WHO recommended intake of 400 g/d, affordability, food safety, home production, variety, and seasonality, and campaigns were carried out through radio stations, primary health care centers, religious centers, and schools.

All 3 interventions targeted consumers and FV vendors within the selected study areas. Therefore, the first intervention at the vendor level and the promotional campaign targeted all selected respondents. In contrast, the coupon system followed a randomized controlled trial design with part of respondents receiving the intervention (coupons) and others not (control group). A total of 600 Vietnamese and 610 Nigerian females were included at the FVN baseline, which decreased to 494 Vietnamese and 473 Nigerian at the end of the study. The main reasons for loss to follow-up were unwillingness to continue, unavailability at the time of interviews, or migration outside the study area.

### Ethical approval

Ethical approvals for the aforementioned research project were obtained prior to the start of the study from Hanoi Medical University Institutional Review Board in Hanoi (45-18/HMU-IRB) and University of Ibadan/University College Hospital Ethical review Committee (UI/UCH-ERC) in Nigeria (HNHREC/05/01/2008a), and the International Food Policy Research Institute’s Institutional Review Board (IFPRI IRB-007490). The randomized controlled trial associated with the affordability intervention was registered with the American Economic Association’s registry (AEARCTR-0007701). All participants signed an informed consent before the start of the study and confirmed the consent by telephone before the subsequent data collection rounds.

### Study design and dietary assessment

The study was designed as a panel and participants were followed for 1 y. Dietary intake data was collected every 2 mo for a total of 6 timepoints (T1–T6). The first assessment (T1) was performed before the start of the interventions; T2, T3, T4, and T5 during the interventions; and T6 after the interventions. Dietary assessment at T6 was performed 3 mo after the end of the interventions in Vietnam and immediately after the end of the interventions in Nigeria. The 3-mo delay of data collection faced in Vietnam was because the planned home visits were restricted by governmental directives imposed from July to September 2021 to limit the spread of COVID-19. Data on FV intake were measured in Vietnam between 28 July, 2020 and 27 September, 2021 and in Nigeria between 24 November, 2020 and 15 December, 2021. Data were collected with a DQQ, a simple, relatively quick method and of low burden for interviewers and participants [21]. The questionnaire consists of 29 dichotomous questions (yes/no) on the food groups consumed the previous day, including a list of country-specific sentinel food items within the same food group. The DQQ was administered as part of a larger survey at T6, but it was administered in the first module to minimize any potential effects of survey fatigue, which could cause differences between answers at T6 and T1 through T5. Additionally, a questionnaire was administered at T1 to obtain sociodemographic information and at T6 to assess the self-reported exposure to the interventions. The latter included multiple-choice questions about each intervention. Respondents were asked to report whether they noticed or used specific components of the interventions, showing supporting images of the interventions. Due to the governmental restrictions to limit the spread of COVID-19, data were collected via phone and, when possible, in-person interviews performed by local researchers using digital forms in KoboToolbox software [31] in both countries.

### Variables

The 6 FV groups (*dark green leafy vegetables, vitamin A-rich orange vegetables, other vegetables, vitamin A-rich fruits, citrus, other fruits)* from the DQQ were used to create the FV score as the main outcome. The FV score ranges from 0, meaning no FV groups consumed during the previous day, to 6, indicating all FV food groups were consumed. It was assumed that a higher score indicated a higher and more diverse intake of FV at population level [22]. For individual FV groups, a dichotomous score (0–1) was created to indicate whether the food group was consumed or not at each timepoint. Based on the self-reported information, respondents were categorized into 4 groups according to exposure to the interventions in the previous year: *not exposed* (0), *exposed to 1 intervention* (1), *exposed to 2 interventions* (2), and *exposed to all interventions* (3). As the degree of exposure was assessed only at T6, the association between FV score and exposure to the interventions was investigated only with data from respondents who were interviewed at T6.

### Data analysis

Data was first explored with descriptive statistics for sociodemographic information at baseline and FV food groups consumed at each timepoint. Potential confounders and effect modifiers were identified for all studied associations. The confounders assessed were *1*) area, age, and household size because FV intake might vary based on individual and household characteristics [26]; *2*) baseline FV score because it influences the possible changes in consumption; and *3*) education, occupation, and food insecurity because underprivileged females possibly have a lower FV intake [32,33]. These indicators were also studied at each timepoint to check for confounders that could have been introduced by loss to follow-up [34]. The only effect measure modifier that was assessed was area since availability and accessibility of FV groups and exposure to the interventions could vary between locations [35]. As area was found to be an effect measure modifier in all models for Vietnam, we decided to analyze data separately for urban and periurban areas for both countries.

The change in the total FV score (ranging from 0 to 6) at population level over the 6 timepoints was analyzed with a generalized Poisson regression, which was selected because the count data were found to be underdispersed [36]. Timepoints were included in the model as independent variables and the FV score as a dependent variable. A random intercept and random slope were added to fulfill the assumption of independence of measurements within persons.

The changes in the probability of consumption of individual FV groups were analyzed over time with mixed effects logistic regression models. Having consumed a specific FV group or not on the previous day was the dependent variable of each model, timepoints were the independent variables, and estimated coefficients reflected probabilities of consumption.

For both analyses, measurement dependency was assessed by likelihood ratio tests and intraclass correlation coefficients (ICCs). A random intercept and random slope were added to correct for the measurement dependency only in the models with ICC >0.05 [37,38]. For these models, the differences between the model with or without random intercept and random slope were checked. If no difference was found, the simplest model was kept.

To study the association of exposure to the interventions and the FV score, we developed a count model with exposure to intervention as independent variable and FV score as dependent variable. FV scores at T5 and at T6 were compared to the degree of exposure to the interventions. T5 was chosen because participants were most likely to have been exposed to the interventions during the previous year, and T6 provided information on the lasting effect on FV score after the interventions. Area was included as covariate only in the models of Vietnam as this was found to be associated with exposure to the interventions and FV score. For each model, all covariates were tested, and the final model was selected based on the lowest Akaike Information Criterion and Bayesian Information Criterion. Data analysis was performed with Stata [39] software and performed separately for Vietnam and Nigeria.

## Results

### General characteristics

In Vietnam, half of the participants were from Đống Đa (50%), and the mean age of the study population was 35 (8.2) y (Table 1). On average, females lived in a household of 5 people, had 2 children, and were married (92%). Females were mainly employed with a regular salary (44%), and in Hà Đông, more people were employed in *crop production and livestock raising* compared to Đống Đa (14% and 0.3%, respectively). In general, most participants *finished high school* (33%), but participants in urban areas were more likely to be higher-educated.

**TABLE 1:** Sociodemographic characteristics of the study population of Hanoi, Vietnam and Ibadan, Nigeria at baseline measurement per area

Distribution over areas	Vietnam			Nigeria		
	Hanoi (total)	Đống Đa (urban)	Hà Đông (periurban)	Ibadan (total)	Abàeja (urban)	Bagadajé (periurban)
***n***	600	297	303	610	296	314
**Urban area , % (*n*)**	49.5 (297)	100 (297)	0	48.5 (296)	100 (296)	0
**Age, mean ± SD**^1^	35.1 ± 8.2	35.2 ± 8.5	35.1 ± 7.9	34.7 ± 8.3	34.7 ± 8.4	34.7 ± 8.2
**Household size, mean ± SD**^2^	4.8 ± 1.9	4.9 ± 1.5	4.7 ± 2.3	5.2 ± 2.0	4.9 ± 1.9	5.4 ± 2.1
**Number of children, mean ± SD**^1^^,^^2^	2.0 ± 0.9	2.2 ± 0.9	1.7 ± 0.8	2.8 ± 1.8	2.6 ± 1.8	3.1 ± 1.7
**Main occupation, % (*n*)**^3^						
Crop production/livestock	7.2 (43)	0.3 (1)	14.0 (42)	0.3 (2)	0.7 (2)	0.0 (0)
Trading	11.9 (71)	13.8 (41)	10.0 (30)	52.1 (318)	48.0 (142)	56.1 (176)
Salary employment	44.4 (265)	52.2 (155)	36.7 (111)	10.5 (64)	11.8 (35)	9.2 (29)
Non-agriculture daily laborer	16.3 (97.3)	8.4 (25)	24.0 (73)	0.0 (0)	0.0 (0)	0.0 (0)
Unpaid housework	5.9 (35)	6.4 (19)	5.3 (16)	1.3 (8)	0.7 (2)	1.9 (6)
Artisan/handicraft	0.0 (0)	0.0 (0)	0.0 (0)	26.2 (160)	25.3 (75)	27.1 (85)
Other	14.4 (86)	18.9 (56)	10.0 (30)	9.5 (56)	13.5 (40)	5.7 (18)
**Highest education level, % (*n*)**^1^^,^^2^						
Primary school	4.9 (29)	1.4 (4)	8.4 (25)	19.3 (118)	16.6 (49)	22.0 (69)
Secondary school	24.1 (144)	10.4 (31)	37.7 (114)	57.4 (350)	55.7 (165)	58.9 (185)
High school	32.7 (196)	31.3 (93)	34.0 (103)	NA	NA	NA
Tertiary institution	37.4 (224)	55.6 (165)	19.2 (58)	20.8 (127)	24.7 (73)	17.2 (54)
Other	1.0 (6)	1.4 (4)	0.7 (2)	2.5 (15)	3.0 (9)	1.9 (6)
**Marital status, % (*n*)**^1^^,^^2^						
Single	5.7 (34)	7.1 (21)	4.4 (13)	10.0 (61)	12.5 (37)	7.6 (24)
Married, monogamous	91.8 (550)	90.2 (268)	93.3 (277)	77.0 (470)	73.6 (218)	80.3 (252)
Married, polygamous	NA	NA	NA	9.0 (55)	7.5 (22.2)	10.5 (33)
Other	2.5 (15)	2.7 (8)	2.4 (7)	3.9 (24)	6.4 (19)	1.6 (5)

Abbreviations: NA, not applicable; SD, standard deviation.

1 1 missing value in Nigeria.

2 6 missing values in Vietnam.

3 3 missing values in Vietnam.

In Nigeria, 48% of the participants were from Abàeja, and the mean age of the study population was 35 (8.3) y. On average, females lived in a household of 5 people, had 3 children, and were married, either monogamously (77%) or polygamously (9%). Most females had finished *secondary school* (57%), and *trading* was the most dominant employment sector (52%) followed by working in the *artisan/handicraft* (26%) sector.

### Trend of total FV score

**FIGURE 1:**
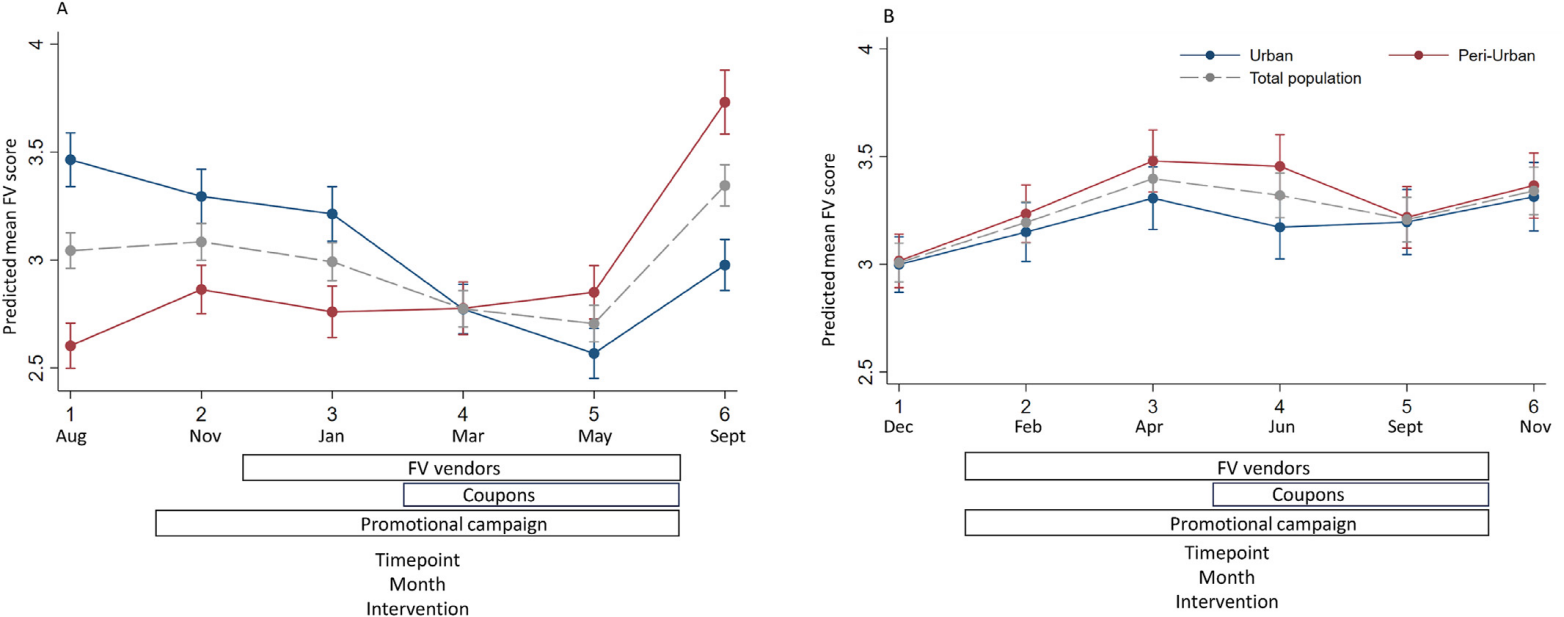
Predicted mean FV score with 95% CI at 6 timepoints of females from (A) Đống Đa (urban) and Hà Đông (periurban), Hanoi and (B) Abàeja (urban) and Bagadajé (periurban), Ibadan. No evidence was found for confounding by area, age, household size, baseline FV score, education, occupation, and food insecurity. Abbreviations: CI, confidence interval; FV, fruit and vegetable.

In Vietnam, Đống Đa had a lower FV score at T1 compared to Hà Đông (3.46; 95% confidence interval [CI]: 3.34, 3.59 compared with 2.60; 95% CI: 2.50, 2.70) (Figure 1A). In Đống Đa, the FV score was relatively stable over time from T1 to T5 and increased by almost 1 point from T5 to T6 (from 2.85; 95% CI: 2.73, 2.97 to 3.73; 95% CI: 3.58, 3.88). In contrast, a downward trend in FV score was shown in Hà Đông from T1 to T5. However, it slightly increased from T5 to T6. At T6, Đống Đa had a higher FV score compared to Hà Đông. In Nigeria, the 2 areas followed a similar trend in mean FV scores over time (Figure 1B). The FV score was relatively stable over all timepoints and ranged from 3.00; 95% CI: 2.87, 3.13 to 3.48; 95% CI: 3.34, 3.62. In both areas, a small increase in the mean FV score was observed between T1 and T3. Moreover, the FV scores at T6 were slightly higher compared to T1 in both areas.

### Trend of single FV groups

**FIGURE 2:**
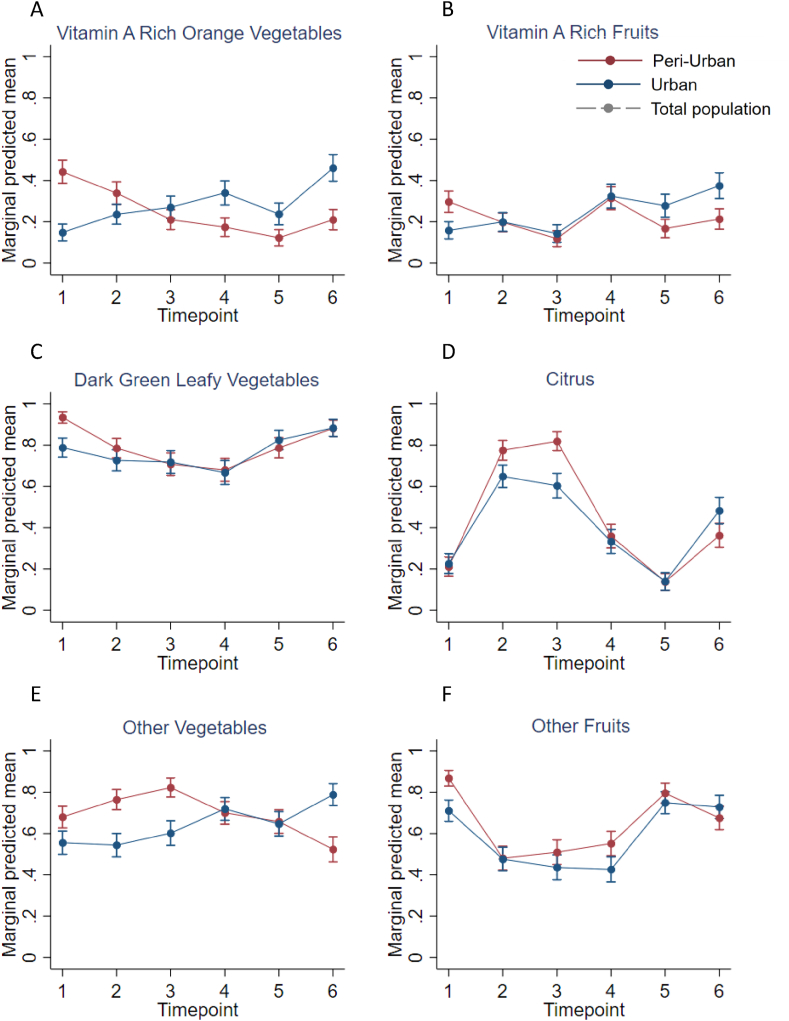
(A–F) Mean probabilities of having consumed individual FV groups at each timepoint with 95% CI in Hanoi, Vietnam for Đống Đa (urban) and Hà Đông (periurban). Timepoint 1 = before the interventions, timepoint 2–3 = 2 interventions implemented, timepoint 4–5 = 3 interventions implemented, and timepoint 6 = after the interventions. Abbreviations: CI, confidence interval; FV, fruit and vegetable.

The trend of consumption over time differed for the individual FV groups, and differences were observed between areas in the Vietnamese population (Figure 2A–F). In general, low consumption of *vitamin A-rich orange vegetables* was observed, and the probability of consumption increased over time for Đống Đa, whereas it decreased for Hà Đông. The probability of consumption of *dark green leafy vegetables* was high and stable throughout the year for both areas. The trend of consumption observed for the *other vegetables* was stable from T1 to T5. However, it increased at T6 in Đống Đa, whereas in Hà Đông it increased at T2 and T3 but decreased afterwards with the lowest probability at T6. The probability of consumption of *vitamin A-rich orange vegetables* was low all year round but increased over time for Đống Đa, with probabilities twice as high at the last 3 timepoints, whereas in Hà Đông, the highest probabilities were observed at T1 and T4. A large variation in consumption levels over time was shown within the *citrus* group. The highest probabilities were observed at T2 and T3, which were 3 to 4 times higher than T1 and T5 in both areas. Large variation over time was also shown for the *other fruit* group with the highest probabilities of consumption at T1, T5, and T6 in both areas.

**FIGURE 3:**
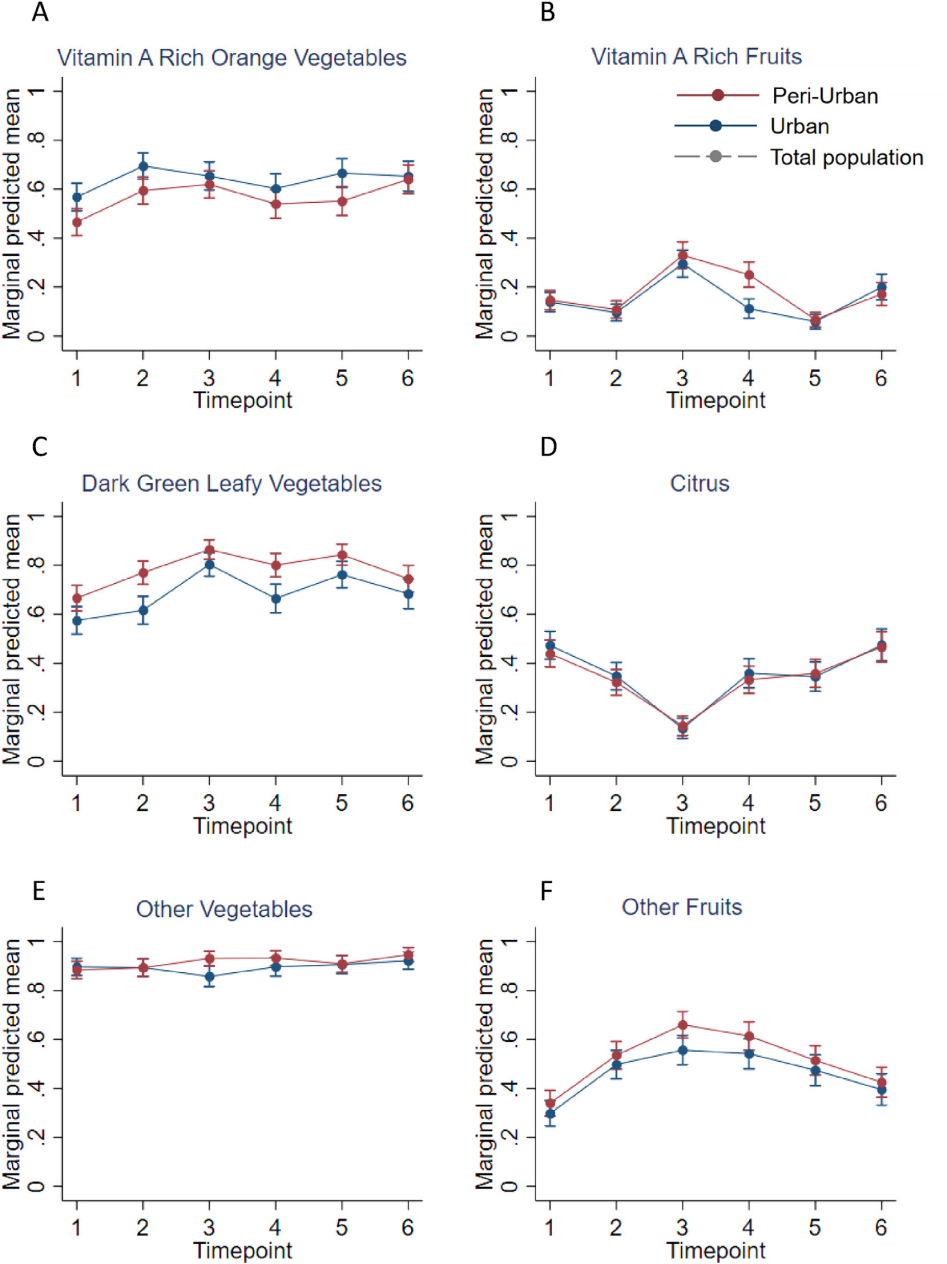
(A–F) Mean probabilities of having consumed individual FV groups at each timepoint with 95% CI in Ibadan, Nigeria for Abàeja (urban) and Bagadajé (periurban). Timepoint 1 = before the interventions, timepoint 2–3 = 2 interventions implemented, timepoint 4–5 = 3 interventions implemented, and timepoint 6 = after the interventions. Abbreviations: CI, confidence interval; FV, fruit and vegetable.

In Nigeria, the trend of consumption over time differed for the individual FV groups, but they were similar in the 2 areas (Figure 3A–F). The probability of consumption for the 3 vegetable groups was high and stable. A small increase was observed in the probability of consumption of *vitamin A-rich orange vegetables* from T1 to T2 and of *dark green leafy vegetables* from T1 to T3, with a small decrease from T3 to T6. The probability of consumption of the *other vegetables* group was the highest and the most stable over time. The probability of consumption of *vitamin A-rich fruits* was low all year round except from T2 to T3 when consumption doubled. Most variation over time was observed for *citrus* and *other fruits* groups. The probability of consumption of *citrus* dropped at T3 to a level more than twice as low as T1; it increased between T3 and T6 reaching the same level as T1. In contrast, the *other fruits* group showed an upward trend between T1 and T3. First, the probability almost doubled, and then it decreased between T3 and T6.

### Association between exposure to the interventions and FV score

**FIGURE 4:**
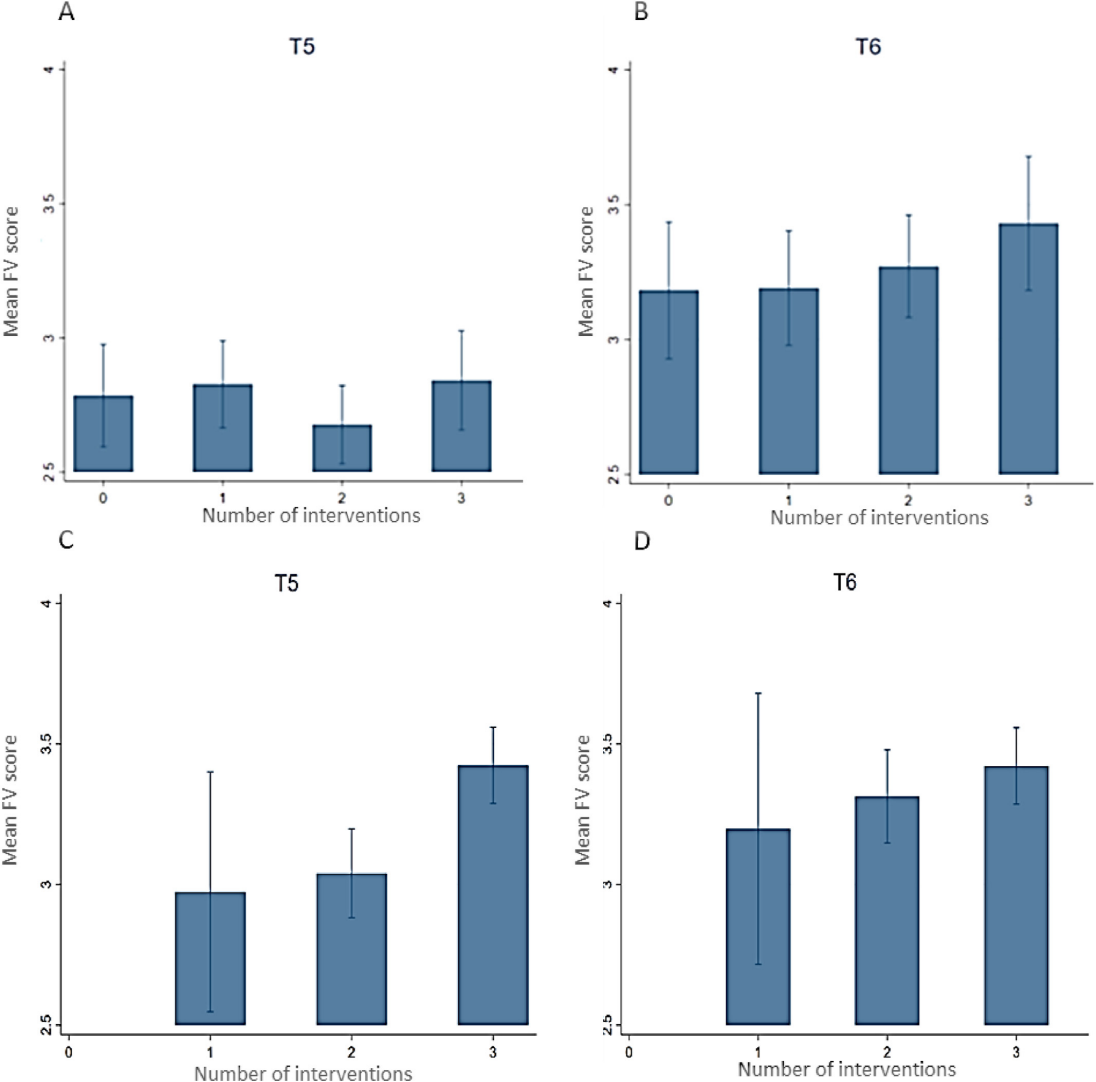
Mean FV score with 95% CI compared between exposure groups at T5 (*n* = 521 Vietnam; *n* = 505 Nigeria) and T6 (*n* = 494 Vietnam; *n* = 473 Nigeria) for Vietnam (A–B) and Nigeria (C–D). 0 = not exposed, 1 = exposed to 1 intervention, 2 = exposed to 2 interventions, 3 = exposed to all interventions. The model was adjusted for area in Vietnam. FV score of the nonexposed could not be calculated as all participants were exposed to at least one intervention. Abbreviations: FV, fruit and vegetable; T, timepoint.

In the total of 494 Vietnamese participants interviewed at T6, 18.4% of the population reported not being exposed while 26.7%, 34.0%, and 20.9% reported being exposed to 1, two 2, or 3 interventions, respectively. The mean FV scores of the exposure groups were relatively similar but slightly higher at T6 compared to T5, ranging from 2.65; 95% CI: 2.50, 2.79 to 2.85; 95% CI: 2.68, 3.0 at T5, and from 3.11; 95% CI: 2.87, 3.34 to 3.46; 95% CI: 3.22, 3.70 at T6 (Figure 4A–D). In the total of 473 Nigerian participants interviewed at T6, all reported being exposed, of which 4.2%, 37.2%, and 58.6% of the population were exposed to 1, 2, or 3 interventions, respectively. The mean FV scores of the exposure groups ranged from 2.97; 95% CI: 2.55, 3.40 to 3.42; 95% CI: 3.29, 3.56 at T5 and from 3.20; 95% CI: 2.72, 3.68 to 3.42; 95% CI: 3.29, 3.56 at T6. No large differences were observed in mean FV score between the exposure groups both at T5 and T6.

## Discussion

In this study, we investigated the consumption trend of total and single FV food groups during the FVN project in urban and periurban Vietnamese and Nigerian females. While the total FV consumption did not vary strongly over the intervention period in either country, the intakes of single FV groups, especially fruits, fluctuated over time. In Vietnam, we also found differences in FV consumption and changes herein between urban and periurban areas.

In both countries, we found that the total FV score remained stable over the study period, with only a slight increase in both countries. This finding suggests that the number of females consuming FV and the diversity and variety of FVs consumed did not drastically change over the intervention period.

It is uncertain whether this finding indicates that the quantities consumed were stable over time, because the FV score on the DQQ does not directly capture information on the quantities consumed since only consumption (yes/no) of FV food groups is reported. A validation of the FV score previously carried out in the same population in the FVN project by the authors showed that a higher FV score was correlated with a higher FV intake, stronger in Nigeria (β = 0.62, *P* < 2e-16) than Vietnam (β = 0.21, *P* = 60.4e-14) [22]. In settings where a low proportion of females consume FV, an increase in this proportion would indicate an increase in FV intake [40]. However, in settings where FV are commonly consumed by most of the females but in inadequate amounts, the FV intake can only be improved by increasing portion sizes. In the first setting, the FV score will capture the change, but in the latter, it will not be able to reflect changes in intake. This could also explain the stable score of vegetable intake as on average 98% and 96% of the population consumed vegetables in Vietnam and Nigeria, respectively. For fruits, in both countries, these percentages were lower (62% in Vietnam and 71% in Nigeria), and hence, the fluctuation may better reflect the changes in amounts of fruit consumed.

Contrary to the relative stability of total FV score, we did see large variation in consumption of individual FV food groups over the year and more for fruits than for vegetables in both countries. These trends largely followed the seasonal availability of FV, a major determinant of consumption in a population that relies on short food chains, where availability, diversity, and affordability are shaped by the seasons [41]. The consumption of *citrus fruits* during the dry season and *vitamin A-rich fruit and vegetables* and *other fruits* during the wet season follows the peaks in availability of these fruits [27,28]. The FV score is indeed a suitable indicator to detect seasonal fluctuation of consumption because of its dichotomous nature. A higher score reflects the consumption of the food group, implying that specific FV are available and accessible at a certain time of the year. According to a preliminary cross-sectional study by Herforth et al. [40], as the FV component of the DQQ positively correlates with FV consumption, the fluctuation of the FV score in our study shows periods of low and high intake of fruits through the year.

In Vietnam, we found an increased intake trend of total FV in the periurban area but not in the urban area. This was mainly due to an increase in the proportion of females reporting consumption of *vitamin A-rich fruits, vitamin A-rich orange vegetables*, and *other vegetables*. Although we do not have primary data on participants’ own FV production in our study population, the difference between periurban and urban areas could be partially explained by the production of FV by households in the periurban area. As Hà Đông was recently added to the city boundaries, several periurban households still have vegetable and fruit tree gardens used for household consumption [42]. This production could have also contributed to maintaining consumption during the intervention period, which was characterized by disruptions of transportation and markets, fluctuation of prices, and limited mobility due to the COVID-19 pandemic [43]. Moreover, limited access to wet markets in urban areas and widespread absence of storage facilities for fresh foods may have affected food choices and consumption [44]. Availability of one’s own produce might have mitigated these effects in periurban areas while households in urban areas might have been compelled to reduce their FV consumption [44].

Due to the nonrandomized placement of 2 of the 3 interventions and the absence of a control group, we cannot attribute changes in our outcome variables to the interventions. These prevented controlling for the effect of temporal factors influencing the study outcome other than the interventions, such as COVID-19 measures put in place to limit the spread of the pandemic. We may speculate that being exposed to the interventions protected females from COVID-19-related disruptions in the food system, possibly leading to a decrease in FV consumption. Some studies are indicating this negative effect on diet quality [45,46] while others suggest an increased intake because of the believed boosted immunity [[47], [48], [49]]. However, the design of and data available from our study do not allow us to test this hypothesis directly.

To note an effect, we associated the FV score with the degree of exposure to the interventions, i.e., having been involved in 1, 2, or 3 of the interventions. This was based on self-reported experienced exposure, which might have been underreported as the promotional campaign in the market environment and local vendors could have been unconsciously experienced by the respondents but not reported. In addition, the loss to follow-up of 20% could have introduced a selection bias at T5 and T6. People exposed to the interventions may have been more likely to stay involved in the study because they were more aware of the benefits of FV. However, sociodemographic characteristics and the baseline FV score of people lost to follow-up were comparable to the participants that were involved until the end.

Overall, monitoring the program outcomes over the period of the interventions allowed identification of the direction and trend of FV intake. Although the effect of the interventions on consumption needs to be interpreted with caution due to the abovementioned limitations in the study design, absence of information on participants’ own FV production, especially in the periurban areas, and the multifactorial nature of consumption, this study highlights the relevance of a comprehensive approach of simultaneously addressing multiple causes of low FV consumption through 3 bundled food system interventions. In this study, the FV score was selected as indicator because it is simple to administer, quick, and relatively cheap. Although it did not directly provide information about FV quantities, it is a proxy for quantities and captures other aspects of FV intakes, such as diversity, variety, and fluctuation over the seasons. Additionally, the use of FV score and count modeling allowed comparison across different contexts and a broader outcome range compared to a binary score. Furthermore, the longitudinal study design is appropriate for monitoring nutrition interventions and capturing seasonal effects. Lastly, implementation of the interventions in an urban and a periurban area of 2 different countries provided accurate insights of FV intakes in different settings and contexts.

In conclusion, we found a marginal increase in the proportion of urban females consuming FVs during the interventions in Vietnam and Nigeria. The FV score appeared to be a simple, quick, and easy-to-use indicator for monitoring diversity, variety, and consumption.

## Author contributions

The authors’ responsibilities were as follows—GP, IDB, LTH, TTM, FOS, EFT: designed research; GP, MS, XTT, FOS, OFS, TEE, BE, RH, ML, AB, SW, KA, GM: conducted research; GP, MS, HV: analyzed data; GP: wrote the first version of the paper; IDB, MS, HV, FOS, OFS, TEE, BE, ML, AB, SW, KA, GM, ADF, EFT: commented on subsequent versions of the paper; GP, IDB, EFT: had primary responsibility for final content; and all authors: read and approved the final manuscript.

## Conflict of interest

The authors report no conflicts of interest.

## Funding

This work was undertaken as part of the CGIAR Research Program on Agriculture for Nutrition and Health (A4NH), with support from the CGIAR Trust Fund. Data was collected under the Fruit and Vegetable Intake in Vietnam and Nigeria (FVN) project funded by the Bill and Melinda Gates Foundation (BMGF) and the UK Foreign, Commonwealth & Development Office (FCDO), project nr OPP1182727. The funders had no role in the design of the study; in the collection, analyses, or interpretation of data; in the writing of the manuscript, or in the decision to publish the results.

## Data availability

Data described in the manuscript, code book, and analytic code will be made available upon request pending PhD thesis dissertation publication.
